# Weighted risk scoring system for predicting peripartum transfusion: development and internal validation

**DOI:** 10.3389/fmed.2026.1746669

**Published:** 2026-05-21

**Authors:** Gökçenur Karakelleoğlu, Elif Ceren Nur Kırımlı Yanık, Batuhan Üstün, Nuh Mehmet Erbakırcı, Eray Çalışkan

**Affiliations:** 1 Department of Obstetrics and Gynecology, Faculty of Medicine, Istanbul Okan University, Istanbul, Türkiye; 2 Department of Obstetrics and Gynecology, Faculty of Medicine, Namık Kemal University, Tekirdağ, Türkiye; 3 Kütahya Şehir Hastanesi, Kütahya, Türkiye; 4 Private Clinic, Istanbul, Türkiye

**Keywords:** blood transfusion, bootstrap method, logistic models, obstetrics, receiver operating characteristic curve, risk assessment, risk factors, validation studies as topic

## Abstract

**Background:**

Early identification of obstetric patients at risk for transfusion can improve perioperative planning and resource allocation. This study aimed to develop and internally validate a weighted scoring system using routinely available pre-delivery parameters.

**Methods:**

We retrospectively analyzed deliveries in a tertiary center between [June 2020–June 2025]. Three continuous variables—platelet count, hemoglobin, and prothrombin time—were dichotomized using receiver operating curve characteristic (ROC)–derived thresholds. These, along with placenta previa, emergency cesarean delivery, and known coagulopathy, entered multivariable logistic regression. Model coefficients determined the Weighted Transfusion Risk Score (0–10). Discrimination was assessed by area under the ROC curve (AUC) in derivation (70%) and validation (30%) cohorts, with bootstrap resampling.

**Results:**

Among 249 women, 69 (27.7%) required transfusion. Six variables remained significant predictors: placenta previa, emergency cesarean delivery, prolonged PT, low hemoglobin, low platelet count, and coagulopathy. The weighted transfusion risk score achieved an AUC of 0.838 (95% CI 0.784–0.892) overall, with 92.8% sensitivity and 57.2% specificity at the optimal cut-off. Validation yielded similar discrimination (AUC 0.871).

**Conclusion:**

A weighted score based on common clinical and laboratory data effectively stratified transfusion risk and retained performance on internal validation. Incorporation into standardized hemorrhage protocols may enable earlier recognition of high-risk patients, more efficient mobilization of resources, and targeted preventive measures. External validation is needed to confirm applicability.

## 1 Introduction

Postpartum hemorrhage (PPH) remains a leading cause of maternal morbidity and mortality worldwide (1, 2). Blood transfusion is a critical component in the management of severe PPH, yet inappropriate delays or under-preparation can compromise patient outcomes. Several transfusion risk-prediction models have been proposed, ranging from expert-based instruments such as the California Maternal Quality Care Collaborative (CMQCC) tool to regression-based calculators (3, 4). However, many of these approaches demonstrate limited discrimination or lack feasibility for routine bedside use. For example, a recent multicenter U. S. study introduced a logistic regression–based numeric risk score that achieved an area under the curve (AUC) of approximately 0.80, but its operational complexity limited practical adoption (5).

More recently, machine learning–based methods, including gradient boosting models, have shown promising predictive accuracy for transfusion during cesarean delivery, with AUC values exceeding 0.80 (6). Nonetheless, such algorithms often lack transparency and remain difficult to integrate into standard clinical workflows.

There is therefore an unmet need for a simple, interpretable tool that leverages readily available preoperative clinical and laboratory parameters, applies a weighted scoring system derived from regression coefficients, and undergoes rigorous internal validation. Weighted, point-based models can combine predictive performance with clinical practicality (7).

In this study, we developed and internally validated a Weighted Transfusion Risk Score for obstetric patients, incorporating platelet count, hemoglobin, and prothrombin time alongside key clinical predictors such as placenta previa, emergency cesarean delivery, and known coagulopathy. The score was tested in derivation and temporal validation cohorts and evaluated using bootstrapping to confirm stability. Our aim was to provide a robust yet user-friendly risk stratification tool to aid in anticipating transfusion needs in obstetric practice.

## 2 Materials and methods

### 2.1 Study design and setting

This retrospective cohort study was conducted at a single tertiary obstetric referral center. Ethical approval was obtained from the institutional review board (approval number: [E-10840098-202.3.02-4878], date: [29.07.2025]), and the study was carried out in accordance with the principles of the Declaration of Helsinki. Informed consent not required due to retrospective design; institutional approval obtained.

### 2.2 Participants

All cesarean deliveries between June 2020 and June 2025 were reviewed. Only patients undergoing cesarean delivery were included in the analysis. Although vaginal delivery was initially considered during study design, no patients meeting the study criteria were identified in this group. Women with a gestational age of ≥24 weeks and complete preoperative laboratory and clinical data were eligible. Among these, 401 patients with complete clinical and laboratory data were identified and screened for eligibility. Of these, 152 were excluded: 38 deliveries occurred outside the facility, 112 had missing essential clinical or laboratory records (15 of whom had received a transfusion), and 2 patients had sickle cell disease while 1 had *β*-thalassemia major—conditions that were not predefined as predictor variables. The final study population comprised 249 women, of whom 69 (27.7%) required peripartum transfusion.

### 2.3 Data collection and variables

Demographic, obstetric, and laboratory information was retrieved from the hospital’s electronic records. The data were accessed for research purposes on 10 January 2024. All patient identifiers were removed before analysis, and the researchers did not have access to any information that could identify individual participants during or after data collection. The following parameters were collected and defined:

- Maternal and obstetric characteristics: age, parity, type of cesarean delivery (emergency vs. elective), multiple gestation, placenta previa, fibroid presence, history of pre-existing coagulopathy, use of low-dose aspirin (ASA) or low-molecular-weight heparin (LMWH), and anesthesia type (general or regional).
- Placenta previa: diagnosis confirmed by ultrasonography in the third trimester, with placental tissue partially or completely covering the internal cervical os.
- Coagulopathy: Defined as a documented pre-existing diagnosis of thrombocytopenia (<100 × 10^3^/μL), von Willebrand disease, or any inherited/acquired coagulation factor deficiency recorded in the patient’s medical history. All patients underwent standard preoperative coagulation testing (platelet count, PT, aPTT, INR). Specific assays such as von Willebrand factor levels or coagulation factor studies were performed only in patients with a prior history or clinical suspicion of a bleeding disorder. Coagulopathy status was therefore determined from confirmed diagnoses rather than universal laboratory screening. ASA use: use of low-dose acetylsalicylic acid during the index pregnancy and continued until delivery or discontinued within ≤7 days before delivery.
- LMWH use: administration of prophylactic or therapeutic low-molecular-weight heparin during the index pregnancy and continued until delivery or discontinued within ≤24 h before delivery for prophylactic dosing, or ≤48 h for therapeutic dosing.
- Emergency delivery: was defined as emergency cesarean section performed due to maternal or fetal indications.
- Multiple gestation: presence of two or more viable fetuses at ≥24 weeks of gestation.
- Intraoperative data: Operative time was defined as the duration of cesarean section (from skin incision to closure).
- Laboratory variables: preoperative platelet count, hemoglobin concentration, and prothrombin time (PT) were obtained from the last complete blood count and coagulation profile performed within 48 h before delivery. Cut-off values for platelet count (<167.85 × 10^3^/μL), hemoglobin (<12.29 g/dL), and PT (>10.85 s) were determined by receiver operating characteristic (ROC) curve analysis in the full dataset prior to model development.
- Outcome: receipt of any peripartum blood product (red blood cells, platelets, fresh frozen plasma) from delivery until 24 h postpartum.

### 2.4 Institutional transfusion policy

At our institution, decisions regarding peripartum transfusion are based on a combination of clinical assessment and laboratory parameters, in accordance with international obstetric hemorrhage guidelines (ACOG, RCOG, and institutional protocols).

Red blood cell transfusion is generally considered in the presence of ongoing bleeding with hemodynamic instability or when hemoglobin levels fall below approximately 7–8 g/dL, with higher thresholds applied in symptomatic patients or those with comorbidities.

Fresh frozen plasma is administered in cases of coagulopathy, particularly when prothrombin time or activated partial thromboplastin time is prolonged in the setting of active bleeding. Platelet transfusion is considered when platelet counts fall below 50 × 10^3^/μL in the presence of bleeding or prior to surgical intervention.

In all cases, transfusion decisions are made by the attending obstetrician in collaboration with the anesthesiology team, taking into account the overall clinical condition, estimated blood loss, and ongoing hemodynamic status.

Although standardized institutional protocols were in place throughout the study period, individual clinician judgment may have influenced transfusion decisions.

### 2.5 Derivation of predictor cut-off values

Three continuous variables—platelet count, hemoglobin, and PT—were transformed into binary predictors. Cut-off points were determined from ROC curve analysis in the full dataset, yielding thresholds of platelet count <167.85 × 10^3^/μL, hemoglobin <12.29 g/dL, and PT > 10.85 s.

### 2.6 Score development

The three dichotomized laboratory parameters, together with categorical variables (placenta previa, emergency cesarean delivery, and coagulopathy), were entered into a multivariate logistic regression model. Regression coefficients (*β*) from the final model were scaled and rounded to the nearest 0.5 to create weighted points for each variable, so that predictors with larger β coefficients contributed proportionally more to the total score. For example, coagulopathy (*β* = 3.466) contributed 3.5 points, placenta previa (*β* = 2.318) contributed 2 points, and variables with lower β values contributed fewer points. Each variable was entered as a binary indicator (present/absent) according to predefined definitions and thresholds. The total Weighted Transfusion Risk Score ranged from 0 to 10. The optimal cut-off value for the score was determined in the derivation cohort using the maximum Youden Index on the ROC curve, which identified 1.5 points as the threshold for high transfusion risk; this cut-off was applied unchanged to the validation cohort.

### 2.7 Validation strategy

A two-step approach was used to assess model performance:

1) Temporal split validation: cases were ordered chronologically, with the first 70% assigned to the derivation cohort and the last 30% to the validation cohort. The optimal cut-off point—determined by the highest Youden Index in the derivation set—was applied unchanged to the validation set. Discrimination (AUC, sensitivity, specificity) was calculated separately for each cohort.
2) Bootstrap internal validation: to test model stability and account for sampling variability, nonparametric bootstrapping with 1,000 resamples was performed in the full dataset and separately within derivation and validation sets. For each bootstrap sample, cases were drawn with replacement, the logistic regression model was refitted with the total score as the sole predictor, and the AUC was recalculated. Bias-corrected percentile 95% confidence intervals for the AUC were derived from the bootstrap distributions.

### 2.8 Statistical analysis

All analyses were performed using IBM SPSS Statistics (version [27]; IBM Corp., Armonk, NY, USA). Normality of continuous variables was evaluated using the Shapiro–Wilk test. Data were expressed as mean ± standard deviation or median (interquartile range) depending on distribution. Group comparisons were conducted with Student’s t-test or Mann–Whitney U test for continuous variables and χ^2^ or Fisher’s exact test for categorical variables.

Predictors with *p* < 0.10 in univariate analysis were included in the multivariate logistic regression to identify independent associations with transfusion. Model discrimination was quantified by the AUC with 95% CIs, and calibration was examined using the Hosmer–Lemeshow test. A *p*-value <0.05 was considered statistically significant. Multicollinearity was assessed using the Variance Inflation Factor (VIF), and no significant collinearity was observed (all VIF values <5).

### 2.9 Sample size considerations

As this was a retrospective study including all eligible cases during the study period, no *a priori* sample size calculation was performed. However, sample adequacy was evaluated based on the events-per-variable (EPV) principle for logistic regression modeling.

A total of 69 transfusion events were observed, and six predictors were included in the final multivariable model, resulting in an EPV of approximately 11.5. This exceeds the commonly recommended minimum threshold of 10 events per variable, supporting the stability and reliability of the regression model.

Therefore, the available sample size was considered adequate for model development and internal validation.

## 3 Results

### 3.1 Study population

A total of 249 women fulfilled the inclusion criteria, with 69 (27.7%) requiring peripartum transfusion. The baseline demographic, obstetric, and laboratory characteristics of the transfused and non-transfused groups are summarized in Table 1.

**Table 1:** Baseline characteristics of transfused and non-transfused patients.

Variable	Transfused (*n* = 69)	Non-transfused (*n* = 180)	*p*-value
Age (years)	32.5 ± 5.4	31.8 ± 5.7	0.42
BMI (kg/m^2^)	28.1 ± 3.7	27.6 ± 3.5	0.33
Gestational age (weeks)	37.0 ± 1.8	38.0 ± 1.5	0.001
Parity ≥1	45 (65.2%)	110 (61.1%)	0.58
Placenta previa	15 (21.7%)	5 (2.8%)	<0.001
Fibroid presence	8 (11.6%)	12 (6.7%)	0.18
Multiple gestation	5 (7.2%)	9 (5.0%)	0.45
ASA use	10 (14.5%)	15 (8.3%)	0.15
LMWH use	6 (8.7%)	8 (4.4%)	0.20
General anesthesia	25 (36.2%)	30 (16.7%)	<0.001
Operation time (min)	65 (50–80)	60 (45–75)	0.04
Platelet count (×10^3^/μL)	150 (120–170)	180 (160–200)	<0.001
Hemoglobin (g/dL)	11.8 ± 1.5	12.5 ± 1.3	<0.001
PT (s)	11.2 (10.8–11.6)	10.9 (10.6–11.3)	<0.001
aPTT (s)	28.5 ± 3.1	27.8 ± 2.8	0.06
INR	1.05 ± 0.08	1.03 ± 0.07	0.12

ASA: acetylsalicylic acid; LMWH: low-molecular-weight heparin; PT: prothrombin time; aPTT: activated partial thromboplastin time; INR: international normalized ratio.

Data are presented as mean ± standard deviation, median (interquartile range), or number (percentage), as appropriate.

Within the coagulopathy group, 9 patients had thrombocytopenia and 3 had von Willebrand disease.

Among those who received transfusions:

- Red blood cells (RBCs): 53 women were transfused with RBCs, ranging from 1 to 12 units.
- Fresh frozen plasma (FFP): 45 women received FFP, between 1 and 9 units.
- Platelets: 9 women were transfused with platelet concentrates, between 1 and 6 units.
- Combination transfusion: 30 patients received both RBC and FFP. 6 patients received both RBC, FFP and platelets.

### 3.2 Univariate analysis

Univariate comparisons identified significant associations between transfusion and the following variables: placenta previa, emergency cesarean delivery, pre-existing coagulopathy, lower preoperative platelet count and hemoglobin, prolonged PT, fibroid presence, use of general anesthesia, longer operation time, and ASA or LMWH use. These results are presented in Table 2.

**Table 2:** Univariate analysis of factors associated with transfusion.

Variable	OR (95% CI)	*p*-value
Placenta previa	9.48 (3.42–26.29)	<0.001
Emergency cesarean delivery	3.85 (2.10–7.06)	<0.001
Coagulopathy	18.75 (4.02–87.38)	<0.001
Fibroid presence	1.82 (0.77–4.30)	0.16
Multiple gestation	1.47 (0.49–4.43)	0.49
ASA use	1.87 (0.79–4.41)	0.15
LMWH use	1.98 (0.64–6.14)	0.24
General anesthesia	2.93 (1.58–5.44)	<0.001
Operation time >60 min	2.04 (1.09–3.82)	0.02
Platelet count <167.85 × 10^3^/μL	3.01 (1.68–5.38)	<0.001
Hemoglobin <12.29 g/dL	4.25 (2.35–7.70)	<0.001
PT > 10.85 s	5.88 (3.22–10.75)	<0.001

OR: odds ratio; CI: confidence interval; ASA: acetylsalicylic acid; LMWH: low-molecular-weight heparin; PT: prothrombin time.

### 3.3 Multivariate logistic regression

Six independent predictors of transfusion were retained in the final multivariate model: placenta previa (OR 10.160, *p* = 0.039), emergency cesarean delivery (OR 4.133, *p* < 0.001), PT > 10.85 s (OR 3.873, *p* < 0.001), hemoglobin <12.29 g/dL (OR 2.595, *p* = 0.009), platelet count <167.85 × 10^3^/μL (OR 1.696, *p* = 0.163), and coagulopathy (OR 32.032, *p* = 0.003) (Table 3). *β* coefficients from this model were used to construct the Weighted Transfusion Risk Score.

**Table 3:** Multivariate logistic regression model for predictors of transfusion and assigned score points.

Variable	β coefficient	SE	OR (95% CI)	*p*-value	Score points
Placenta previa	2.318	1.121	10.160 (1.121–92.131)	0.039	2
Emergency cesarean delivery	1.419	0.382	4.133 (1.959–8.716)	<0.001	1.5
PT > 10.85 s	1.354	0.371	3.873 (1.857–8.075)	<0.001	1.5
Hemoglobin <12.29 g/dL	0.954	0.366	2.595 (1.272–5.298)	0.009	1
Platelet count <167.85 × 10^3^/μL	0.529	0.380	1.696 (0.804–3.574)	0.163	0.5
Coagulopathy	3.466	1.144	32.032 (3.381–303.408)	0.003	3.5

β: regression coefficient; SE: standard error; OR: odds ratio; CI: confidence interval; PT: prothrombin time.

### 3.4 Score performance in the full cohort

**Figure 1:**
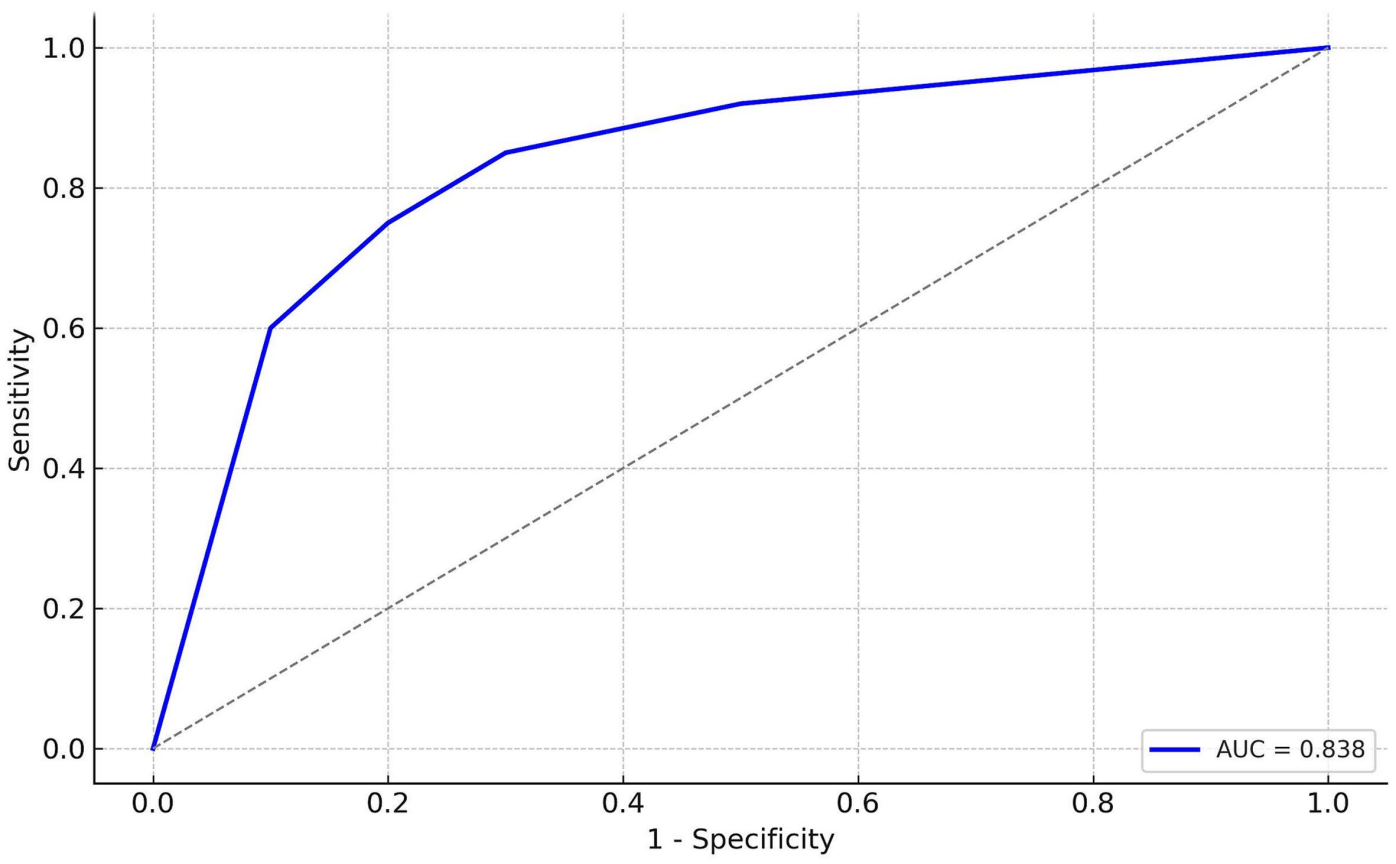
Receiver operating characteristic (ROC) curve for the weighted transfusion risk score in the full study cohort (*n* = 249). The area under the curve (AUC) and 95% confidence interval (CI) are shown. ROC, receiver operating characteristic; AUC, area under the curve; CI, confidence interval.

In the overall dataset, the score yielded an AUC of 0.838 (95% CI 0.784–0.892). Using the optimal cut-off value of 1.5 points, sensitivity was 92.8% and specificity was 57.2% (Figure 1). Consistent with these metrics, observed transfusion rates increased with higher scores (Table 4).

**Table 4:** The transfusion frequency according to the weighted transfusion risk score.

Risk score	Number of patients	Number of transfused patients	Percentage of patients transfused	Total transfusion amount (Units)
0	37	0	0	0
0.5	5	0	0	0
1	31	0	0	0
1.5	40	4	10	13
2	9	1	11	1
2.5	39	14	35	41
3	25	8	32	25
3.5	12	6	50	34
4	23	11	47	29
4.5	13	11	84	49
5	2	2	100	8
6	4	3	75	14
6.5	1	1	100	1
7	2	2	100	3
7.5	2	2	100	5
8	4	3	75	15

### 3.5 Derivation and validation cohorts

Temporal split produced a derivation set of 175 patients and a validation set of 74 patients.

**Figure 2:**
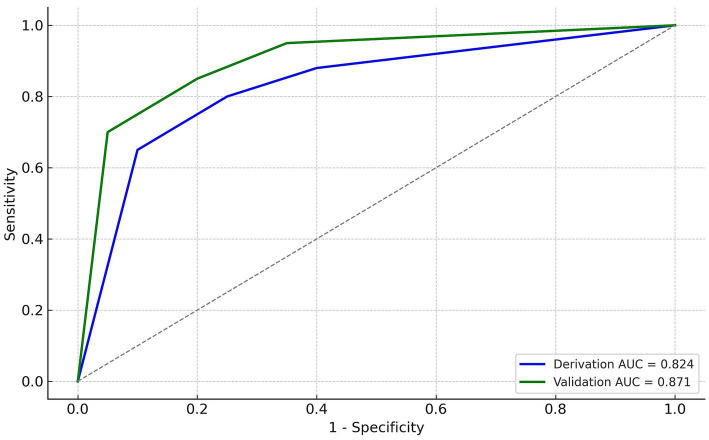
ROC curves for the weighted transfusion risk score in the derivation cohort (*n* = 175) and the validation cohort (*n* = 74). The optimal cut-off value of 1.5 points, determined in the derivation set, was applied unchanged to the validation set. ROC, receiver operating characteristic; AUC, area under the curve; CI, confidence interval.

- Derivation cohort: AUC 0.824, sensitivity 89.6%, specificity 59.5%.
- Validation cohort: AUC 0.871, sensitivity 100%, specificity 51.9%.
  ROC curves for both cohorts are shown in Figure 2.

### 3.6 Bootstrap internal validation

Nonparametric bootstrapping with 1,000 resamples demonstrated stable discrimination:

- Full dataset: bias-corrected AUC 0.840 (95% CI 0.790–0.889)
- Derivation cohort: AUC 0.826 (95% CI 0.759–0.884)
- Validation cohort: AUC 0.872 (95% CI 0.789–0.938)

### 3.7 Score distribution and risk stratification

**Figure 3:**
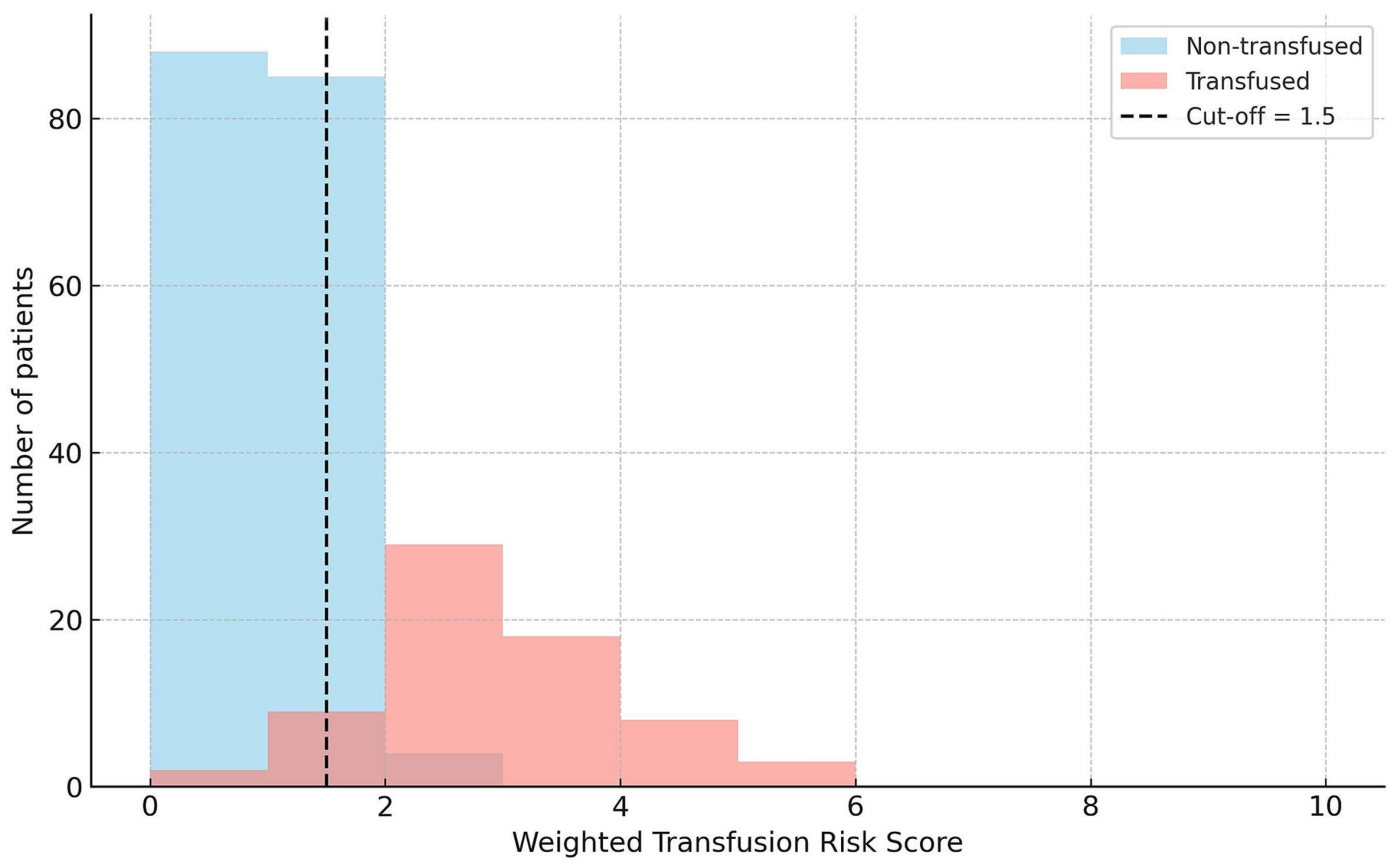
Distribution of the weighted transfusion risk score among transfused and non-transfused patients. The vertical dashed line represents the cut-off value of 1.5 points identified by the Youden index in the derivation cohort.

Distribution of the total score in transfused and non-transfused patients is illustrated in Figure 3. Patients scoring ≤1 point were predominantly non-transfused, whereas those scoring ≥2 points showed a marked increase in transfusion frequency.

**Figure 4:**
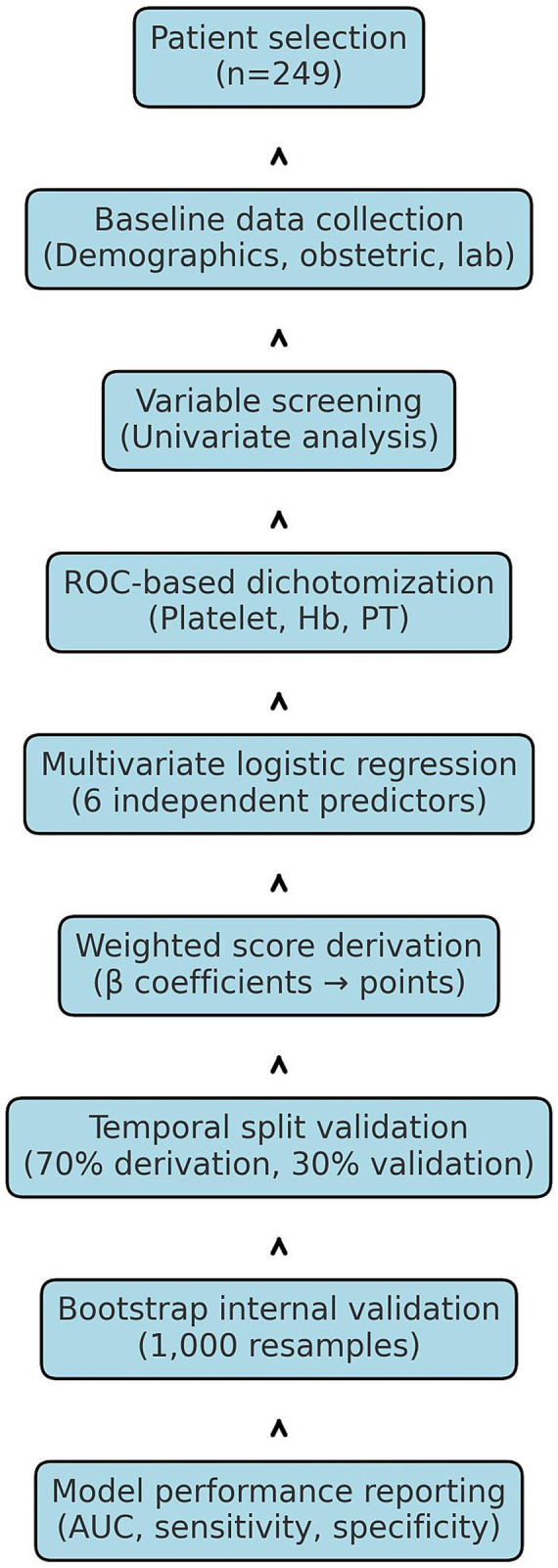
Flowchart summarizing the statistical analysis process. The workflow included patient selection, baseline data collection, univariate analysis, ROC-based dichotomization of continuous predictors, multivariate logistic regression, derivation of the weighted score, temporal split validation, bootstrap internal validation, and performance reporting. ROC, receiver operating characteristic; AUC, area under the curve; CI, confidence interval.

The overall study workflow, including patient selection, data collection, variable selection, model derivation, and validation procedures, is summarized in the flowchart (Figure 4).

## 4 Discussion

In this single-center cohort, we developed a practical model to predict obstetric transfusion using routinely available pre- and intrapartum data. The key predictors identified in our model—placental pathology (e.g., placenta previa), emergency cesarean delivery, prolonged operative time, low baseline hemoglobin, and thrombocytopenia—are consistent with risk factors highlighted in large epidemiological studies and previous transfusion scoring systems (1–6, 8–11). This agreement with established evidence supports the clinical plausibility and potential utility of our model. The transfusion rate in our cohort (27.7%) was higher than typically reported in general obstetric populations, which may be attributed to our institution’s role as a tertiary referral center managing a substantial proportion of high-risk pregnancies, including cases with placenta previa, multiple gestations, and known coagulopathies. Institutional transfusion thresholds and a proactive approach to hemodynamic instability may also have contributed to this higher rate. Hypertensive disorders were evaluated but were not retained in the final model, likely due to their overlap with other clinical predictors and limited independent contribution in this cohort.

Current international guidelines emphasize structured, continuous assessment of hemorrhage risk throughout pregnancy and delivery. The RCOG Green-top 52, ACOG Practice Bulletin No. 183, and FIGO 2022 recommendations all list placenta previa/accreta spectrum, hypertensive disease, and coagulopathy as high-priority conditions for blood product preparedness (8–10). Similarly, the CMQCC Hemorrhage Toolkit operationalizes these into a risk assessment process that is updated intrapartum (11). The overlap between these frameworks and our high-impact predictors suggests that the strength of a data-driven model lies in quantifying individualized probability rather than replacing guideline-based checklists.

Laboratory parameters, particularly platelet count and hemoglobin level, were also significant contributors. This aligns with prior studies showing that low platelet count is associated with higher transfusion rates, even above the threshold for severe thrombocytopenia (12–15). Our ROC analysis suggests that platelet count cut-offs above conventional critical values may still stratify transfusion risk in obstetric populations. Incorporating inflammatory indices such as the systemic immune-inflammatory index (SII), which has been linked to PPH severity in recent studies (15), could further refine predictive accuracy.

Our findings also align with condition-specific models. For example, in placenta previa, Kang et al. demonstrated that combining clinical and ultrasonographic data can predict massive transfusion risk during cesarean delivery with high accuracy (13). While our study included a broader obstetric population, placental pathology remained a dominant predictor, supporting its role as a universal high-risk factor.

Risk prediction models should ultimately improve outcomes, not just statistical metrics. External validations of hemorrhage risk tools have shown benefits as cognitive aids, but highlight the need for balanced sensitivity and specificity (14). Our model’s simplicity—using data readily available before or during delivery—supports integration into existing hemorrhage safety bundles such as those recommended by AIM and CMQCC (11, 12), potentially enabling earlier interventions like tranexamic acid administration, optimized uterotonic use, and rapid escalation when indicated.

Strengths of our work include the focus on an outcome directly relevant to resource allocation (transfusion requirement) and the inclusion of both clinical and laboratory predictors. However, limitations must be acknowledged. First, being a single-center study may limit generalizability, and calibration should be tested in other settings. Second, transfusion decisions may vary between clinicians, though institutional policies remained stable during the study period. These decisions were guided by institutional protocols aligned with international guidelines, although individual clinical judgment remained an inherent component of real-world practice. Third, we lacked standardized imaging markers for placentation and exact quantified blood loss, which could improve predictive performance. Lastly, our sample size limited the ability to assess rarer coagulopathies, though their observed effect was consistent with previous reports (8–10, 15).

In summary, our model supports and quantifies the risk factors emphasized in current guidelines and literature. Future studies should externally validate this approach, compare it with existing tools, and evaluate its integration into structured hemorrhage protocols to determine whether it can reduce morbidity, product utilization, and response times in obstetric hemorrhage management.

## 5 Conclusion

We developed and internally validated a weighted scoring system that combines readily available clinical and laboratory variables to predict obstetric transfusion risk. The model demonstrated strong discriminatory performance and aligns closely with current international guidelines, highlighting its potential for integration into standardized hemorrhage management bundles. By enabling early identification of high-risk patients and timely mobilization of resources, its implementation may improve maternal safety and optimize blood product utilization. External validation across diverse clinical settings is needed to confirm its applicability and impact on outcomes.

## Data Availability

The datasets presented in this study can be found in online repositories. The names of the repository/repositories and accession number(s) can be found at: https://doi.org/10.6084/m9.figshare.30582254.
